# Utilization of cone‐beam CT for reconstruction of dose distribution delivered in image‐guided radiotherapy of prostate carcinoma — bony landmark setup compared to fiducial markers setup

**DOI:** 10.1120/jacmp.v14i3.4203

**Published:** 2013-05-06

**Authors:** Petr Paluska, Josef Hanus, Jana Sefrova, Lucie Rouskova, Jakub Grepl, Jan Jansa, Linda Kasaova, Miroslav Hodek, Milan Zouhar, Milan Vosmik, Jiri Petera

**Affiliations:** ^1^ Department of Medical Biophysics, Faculty of Medicine in Hradec Kralove Charles University in Prague Czech Republic; ^2^ Department of Oncology and Radiotherapy University Hospital Hradec Kralove Hradec Kralove Czech Republic; ^3^ Department of Radiology University Hospital Hradec Kralove Hradec Kralove Czech Republic

**Keywords:** IGRT, cone‐beam CT, prostate margin, fiducial markers

## Abstract

The purpose of this study was to compare two different styles of prostate IGRT: bony landmark (BL) setup vs. fiducial markers (FM) setup. Twenty‐nine prostate patients were treated with daily BL setup and 30 patients with daily FM setup. Delivered dose distribution was reconstructed on cone‐beam CT (CBCT) acquired once a week immediately after the alignment. Target dose coverage was evaluated by the proportion of the CTV encompassed by the 95% isodose. Original plans employed 1 cm safety margin. Alternative plans assuming smaller 7 mm margin between CTV and PTV were evaluated in the same way. Rectal and bladder volumes were compared with initial ones. While the margin reduction in case of BL setup makes the prostate coverage significantly worse (p=0.0003, McNemar's test), in case of FM setup with the reduced 7 mm margin, the prostate coverage is even better compared to BL setup with 10 mm margin (p=0.049, Fisher's exact test). Moreover, partial volumes of organs at risk irradiated with a specific dose can be significantly lowered (p<0.0001, unpaired t‐test). Reducing of safety margin is not acceptable in case of BL setup, while the margin can be lowered from 10 mm to 7 mm in case of FM setup.

PACS numbers: 87.55.dk, 87.55.km, 87.55.tm

## I. INTRODUCTION

Nonpredictable prostate position variation is the challenge for comparison of different IGRT strategies. Techniques of patient setup relative to external beam's isocenter have developed during the last decade. Historically, skin marks and setup lasers have been used. These are not adequate surrogates for prostate position and require extensive safety margins, which are incompatible with the delivery of the high radiation doses above 70 Gy that are currently used in routine practice.^(1)^ Planar X‐ray imaging techniques have enabled registration with skeletal anatomy, but recent studies showed a poor correlation of prostate position and bony anatomy.^(2)^ Prostate location variations were studied relative to the adjacent bony anatomy by Schallenkamp et al.^(3)^ with the conclusion that significant interfractional motion exists between the prostate and the pelvic bony anatomy. These move independently, therefore the pelvic bony anatomy should not be used as a surrogate for prostate motion. The authors also suggest that fiducial markers are stable within the prostate and allow significant margin reduction when used for online localization of the prostate. “The limited interuser variability and the marker stability make markers an ideal surrogate for the prostate position”.^(1)^ While the PTV margin required for bony anatomy alignment should be within the range of 10 mm, the PTV margin reguired for marker alignment can be lowered to under 4 mm.^(4)^ These margins were calculated and were considered as intrafraction motion, which plays only a marginal role in determining PTV margins for bony anatomy alignment, but it does play a crucial role for marker‐based alignment.

Fiducial markers kV imaging versus CBCT for prostate IGRT was compared by Barney et al.^(5)^ They preferred fiducial imaging, because it requires less daily input, is less time‐consuming, and is a more reliable, reproducible treatment. By using fiducials, the uncertainty associated with CBCT soft‐tissue definition can be avoided. A comparison of CBCT automatic grey‐value alignment to implanted fiducial marker alignment was made by Shi et al.^(6)^ with the conclusion that “CBCT with soft‐tissue‐based automatic corrections is not an accurate alignment compared with manual alignment to fiducial markers.” Therefore a daily manual alignment to fiducials is considered to be one of the most reliable methods to maintain accuracy in prostate IGRT.

Substantial positional variation of prostate is caused by a variety of factors. The most significant predictor for intrafraction prostate motion is the status of rectal filling. A full rectal state is invariably associated with mobile gas pockets responsible for the elevated levels of prostate motion. While the apex is largely immobile, prostate motion is well‐described by rotation, but does undergo deformation due to rectal distension.^(7)^ Effects of rectal motion during prostate radiotherapy with regard to rectal dose and clinical target volume (CTV) dose coverage were studied by Sripadam et al.^(8)^ This study revealed instances of insufficient CTV coverage. A large daily variation in rectum and bladder doses based on the anatomy of the day were described in a work by Varadhan et al.^(9)^

IGRT systems provide more information than that which is required for simple patient positioning. Utilization of cone‐beam CT (CBCT) can provide 3D anatomic information directly in irradiation position. Such information enables reconstruction of a current dose distribution. CBCT was evaluated for treatment planning by Yoo and Yin^(10)^ and Yang et al.^(11)^ with the conclusion that CBCT could be used for verification planning to verify treatment delivery retrospectively. There are several recent studies using CBCT for retrospective prostate coverage assessment. Evaluation of the ‘dose of the day’ using post‐treatment CBCT for ten prostate cancer patients with implanted markers was described by van Zijtveld et al.^(12)^ The actual IMRT fluence maps delivered to a patient were derived from measured EPID images acquired during treatment. Eight prostate cancer patients with intraprostatic fiducials were evaluated using weekly CBCT by Pawlowski et al.^(13)^ Twelve prostate patients treated using implanted fiducial guidance were assessed with CBCT scans twice weekly by Hatton et al.^(14)^ We described a group of 17 patients treated with bony anatomy image guidance evaluated using weekly CBCT.^(15)^

The aim of the present study was to utilize the CBCT scans acquired before treatment for dose reconstruction purposes and hereby to compare two different styles of IGRT: bony landmark setup vs. fiducial markers setup. Comparison of isotropic 10 mm and 7 mm margin for both types of image guidance was made.

## II. MATERIALS AND METHODS

### A. Patient characteristics

Fifty‐nine patients with adenocarcinoma of the prostate staged T2a‐T3b N0 M0 treated between August 2008 and December 2010 were evaluated. Patients were treated using intensity‐modulated radiotherapy (IMRT) to prostate with simultaneous integrated boost (SIB) to proximal part of seminal vesicles. Daily image guidance was performed based on two orthogonal kV images: 29 patients using bony landmark (BL) setup and 30 patients using implanted fiducial markers (FM) setup. The clinical and treatment parameters of the two groups are presented in Table 1. The two groups were contemporaneous cohorts. Patients capable and agreeable of implanting fiducial markers were aligned using fiducial markers, while the others were aligned using bony anatomy. In order to assess target volume coverage, CBCT scans were acquired once a week in the treatment position immediately after patient's setup.

**Table 1:** Comparison of clinical and treatment parameters between the patients with bony landmark setup (BL group) and the patients with fiducial markers setup (FM group).

		*BL Group*	*FM Group*
Number of Patients		29	30
Age (years)	mean (SD)	70 (6)	68 (6)
	range	57 – 79	59 – 77
CTV2 (ccm)	mean (SD)	29.8 (11.7)	29.1 (7.3)
	range	16.2 – 58.2	15.5 – 47.2
CTV1‐2 (ccm)	mean (SD)	7.9 (3.2)	6.9 (2.0)
	range	3.5 – 16.8	4.3 – 11.7
CBCT/planning CT volume ratio ‐ CTV2	mean (SD)	0.97 (0.14)	0.99 (0.09)
	range	0.54 – 1.42	0.67 – 1.35
CBCT/planning CT volume ratio ‐ CTV1‐2	mean (SD)	1.01 (0.31)	1.03 (0.30)
	range	0.28 – 1.92	0.50 – 1.97
Rectal volume (ccm)	mean (SD)	58.1 (17.4)	55.4 (16.7)
	range	35.4 – 94.6	33.2 – 103.7
Bladder volume (ccm)	mean (SD)	100 (31.5)	94.5 (34.2)
	range	54.1 – 161.1	51.9 – 208.3
Total number of CBCT scans		134	177
Number of CBCT scans per patient	mean (SD)	5 (2)	6 (1)
	range	1 – 8	3 – 8

### B. Parameters of CT and CBCT

**Figure 1:**
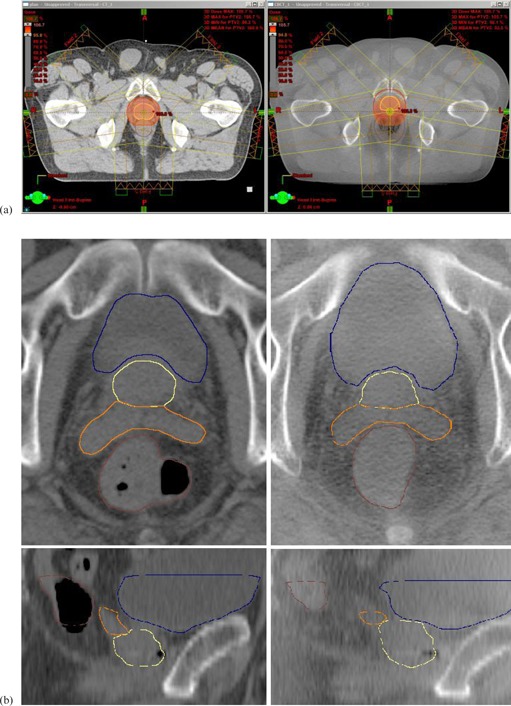
Comparison (a) of planning CT scan (left) and CBCT scan (right) with prostate (CTV2 – yellow line, PTV2 – red line, 1 cm margin) and rectum (brown line) delineated; isocentric transversal slice. Color wash is set to 95% of the dose prescribed to prostate. Using BL‐based alignment, excessive rectal volume causes the prostate movement anteriorly, which leads in prostate underdosage. Lower image quality (b) of CBCT (right) comparing to planning CT (left). Prostate (yellow line), seminal vesicles (orange line), rectum (brown line), and bladder (blue line) are shown in transversal view (upper part) and sagittal view (lower part).

A Siemens Somatom Sensation CT scanner (Siemens Medical Solutions, Erlangen, Germany) was used for acquisition of CT images (512×512 matrix, 0.98 mm pixel size, 3 mm slice thickness). CBCT images were acquired using Varian on‐board imaging system (OBI, Varian Medical Systems, Palo Alto, CA) and reconstructed using about 700 images in a “half‐fan” projection with a bowtie filter acquired over 360° rotation. For CBCT reconstruction, 45 cm diameter and 12 cm axial length with 3 mm slice thickness and 512×512 matrix was used. The technique used was 125 kV, 80 mA, 25 msec. The method to reconstruct the actually delivered dose based on pretreatment CBCT was first validated using phantom measurements. Results were described elsewhere.^(15)^ An example of dose distribution calculated on planning CT and on CBCT is shown in Fig. 1(a), where the BL‐based alignment was used.

### C. Radiotherapy planning and delivery

Intensity‐modulated radiotherapy (sliding window technique) with five coplanar fields to prostate plus proximal two‐thirds of seminal vesicles was planned and delivered. CT slices of 3 mm thickness were acquired. Patients were scanned and treated in a supine position with Dual Leg Positioner (Civco Medical Solutions, Kalona, IA) to immobilize the legs and pelvis.

The patients received instructions for emptying their rectum before the planning CT, as well as before each irradiation session. Patients also obtained glycerin suppositories, but their application was voluntary. Application of suppositories was strongly recommended only when the planning CT had to be repeated by reason of rectum volume. The planning CT scan was repeated in case the rectum volume exceeded 120 cm^3^ (for organs‐at‐risk delineation, see below). Patients were instructed to have slightly filled bladder at time of planning CT. This can be realized by drinking 500 ml of water 45 minutes before planning CT. Instructions for rectum and bladder filling management were similar at time of irradiation.

CT images were transferred to the Eclipse treatment planning system (Varian Medical Systems). Clinical target volumes (CTVs) were delineated as follows: CTV1 represents prostate plus proximal part of seminal vesicles, CTV2 represents prostate alone; thus, CTV1‐2 represents proximal part of seminal vesicles. Then, planning target volumes (PTVs) were created. To obtain the planning target volume for the prostate (PTV2), a 10 mm margin was applied to CTV2 in all directions. PTV1 was constructed in the same manner. PTV1‐2 was obtained by subtracting PTV1‐PTV2. Organs at risk (OARs) — rectum and bladder — were delineated from 1 cm superior to 1 cm inferior to PTV1.

A five‐field intensity‐modulated radiotherapy (IMRT) with simultaneous integrated boost using 6 MV photons was used to deliver 78 Gy to PTV2 and 72.15 Gy to PTV1‐2 in 39 fractions. This regimen corresponds to 2 Gy/fr and 1.85 Gy/fr to prostate and proximal part of seminal vesicles, respectively. The IMRT plan was optimized to fulfill criteria (presented in Table 2) with the PTV2 dose coverage between 95%–96%. In order to evaluate the possibility of margin reduction, alternative plans assuming smaller 7 mm margin between clinical and planning target volume were prepared. These plans were not intended for irradiation, but were made for reconstruction purposes. Alternative plans were optimized for maximal OARs sparing allowed for PTV2 dose coverage between 95%–96% (i.e., comparable coverage to the original plans).

**Table 2:** Prescription doses for planning target volumes and acceptable doses for organs at risk.

*Structure*	*Prescription*
Prostate (PTV2)	Prescribed dose 78 Gy = mean dose for PTV2.
	Minimally 95% of the prescribed dose (i.e., 74.1 Gy) to 95% of the PTV2.
	Maximal dose ≤107% of the prescribed dose (i.e., 83.5 Gy).
Seminal Vesicles (PTV1‐2)	Prescribed dose 72.15 Gy.
	Minimally 95% of the prescribed dose (i.e., 68.5 Gy) to 95% of the PTV1‐2.
	Maximal dose ≤107% of the prescribed dose (i.e., 77.2 Gy).
Rectum	Maximally 50% can receive 50 Gy.
	Maximally 25% can receive 70 Gy.
	Maximally 15% can receive 75 Gy and maximally 15 cm^3^ can receive 75 Gy.
	Maximum dose 78 Gy.
Bladder	Maximally 30% can receive 70 Gy.
	Maximally 15% can receive 75 Gy and maximally 15 cm^3^ can receive 75 Gy.
	Maximum dose 78 Gy.

### D. On treatment CBCT acquisition and assessment

Patients were treated using Varian Clinac 2100C/D linear accelerator equipped with on‐board imager (OBI) kV imaging system version 1.3 with CBCT option. After initial skin marks setup, two orthogonal kV images were acquired and patient's position was corrected based on selected setup strategy, either bony landmark or fiducial markers setup. The patient realignment was performed by the RTT according to the clinical protocol: online registration process with no action threshold. Immediately after setup, CBCT scan was acquired. The resulting images were subsequently sent to the Eclipse treatment planning system, where the treatment isocenter was identified. CTVs and OARs were outlined by a single observer at the same range as on the planning CT. The consistency of these manual contours is illustrated in 2, 3 for prostate and seminal vesicles, respectively. Nominal data can be found in Table 1. CBCT scans were acquired once a week, during treatment fractions no. 2, 5, 10, 15, 20, 25, 30, and 35. CBCTs were not used for setup of the patient, just for retrospective analysis. Original treatment plan based on planning CT was then reconstructed on each CBCT. Reconstruction of the actual delivered dose distribution was performed based on planned fluences and MUs. The isocenter of the reconstructed plan was set to the isocenter of the CBCT scan, which corresponds to online matched treatment isocenter. Alternative plans assuming smaller 7 mm margin were reconstructed in the same way.

**Figure 2:**
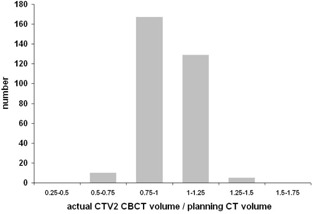
Histogram of actual CTV2 volumes on CBCTs relative to planning CT volumes.

**Figure 3:**
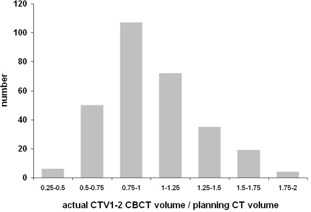
Histogram of actual CTV1‐2 volumes on CBCTs relative to planning CT volumes.

### E. DVH analysis

The reconstructed dose distributions were compared with the planned dose distribution by evaluating the dose‐volume histograms (DVHs) for the prostate (CTV2), seminal vesicles (CTV1‐2), rectum, and bladder. For the prostate and seminal vesicles, the relative volumes that received at least 95% of the prescribed dose were derived. Situations where less than 95% of the CTV was covered by 95% of the prescribed dose were considered as underdosed. Proportion of underdosed plans in each group (BL‐10 mm, BL‐7 mm, FM‐10 mm and FM‐7 mm) was evaluated. These datasets were compared using two‐tailed Fisher's exact test (BL group vs. FM group) and McNemar's test (BL‐10 mm vs. BL‐7 mm group). Rectal and bladder volumes on CBCTs were compared relative to planning CT volumes. Relative rectal volumes and absolute bladder volumes irradiated with doses higher than 75 Gy, 70 Gy, and 60 Gy derived from BL‐10 mm and FM‐7 mm treatment plans were compared using unpaired t‐test.

## III. RESULTS

Number of CBCT scans acquired for one patient during treatment course in BL group was between one and eight, median was five scans. Total number of CBCT acquisitions in BL group was 134. Number of CBCT scans acquired for one patient in FM group was between three and eight, median was six scans. Total number of CBCT acquisitions in FM group was 177. The difference in scanning numbers between the groups is statistically significant (p=0.008, unpaired t‐test). Generally, lower number of CBCT scans than expected was caused by technical problems with the CBCT system or by excessive accelerator workload. Technical problems were the reason especially at the beginning of the study, soon after the installation of the CBCT system. Two patients in BL group (2+3 CBCTs) and two patients in FM group (7+5 CBCTs) were treated without SIB (i.e., only prostate was irradiated as a target volume).

**Figure 4:**
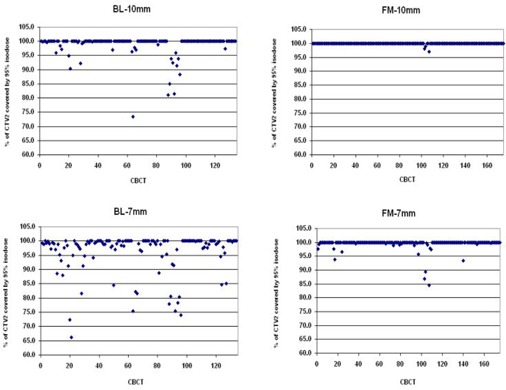
Prostate (CTV2) coverage by 95% of the prescribed dose: the upper part of the figure is for the 10 mm margin and the lower part for the 7 mm margin; bony landmark setup (BL) (left) and fiducial markers setup (FM) (right). The data are grouped by patient.

**Figure 5:**
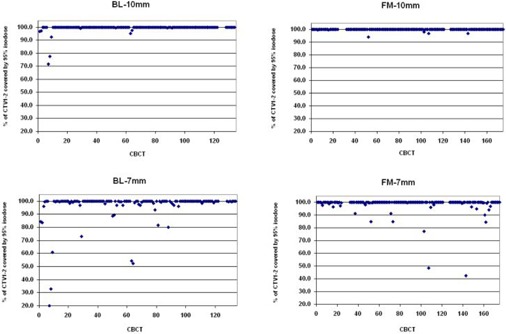
Seminal vesicles (CTV1‐2) coverage by 95% of the prescribed dose: the upper part of the figure is for the 10 mm margin and the lower part for the 7 mm margin; bony landmark setup (BL) (left) and fiducial markers setup (FM) (right). The data are grouped by patient.

In BL group with 10 mm margin (BL‐10 mm), there were 12 of 134 reconstructed plans where 95% of the CTV2 was not covered by the 95% isodose, and these were distributed over five patients. The minimal proportion of CTV2 covered by 95% isodose was 74%, (Fig. 4). Sufficient coverage of CTV1‐2 was not achieved in three of 129 cases, and these were observed within one patient. The minimal proportion of CTV1‐2 covered by 95% isodose was 72% (Fig. 5). Evaluation of alternative plans assuming smaller 7 mm margin (BL‐7 mm group) revealed 27 of 134 cases of CTV2 underdosage distributed over 11 patients, and 13 cases of CTV1‐2 underdosage distributed over eight patients. The minimal proportions of CTV2 and CTV1‐2 covered by 95% isodose were 66% and 20%, respectively. In FM group with 10 mm margin (FM‐10 mm), in all the 177 reconstructed plans the CTV2 was covered sufficiently. Sufficient coverage of CTV1‐2 was not achieved in one of the 165 cases. The minimal proportions of CTV2 and CTV1‐2 covered by 95% isodose were 97% and 94%, respectively. Evaluation of alternative plans assuming smaller 7 mm margin (FM‐7 mm group) revealed six of 177 cases of CTV2 underdosage distributed over four patients, and 11 of 165 cases of CTV1‐2 underdosage distributed over eight patients. The minimal proportions of CTV2 and CTV1‐2 covered by 95% isodose were 85% and 42%, respectively.

**Figure 6:**
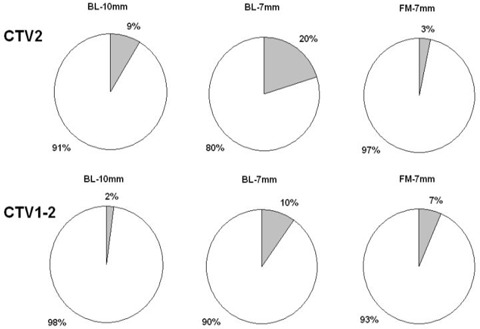
Proportion of plans where prostate (CTV2; upper part) or seminal vesicles (CTV1‐2; lower part) were under dosed (grey shade). Comparison of bony landmark setup with 10 mm margin (BL‐10 mm), bony landmark setup with 7 mm margin (BL‐7 mm), and fiducial markers setup with 7 mm margin (FM‐7 mm).

Numbers of underdosed plans are presented in Table 3. While the margin reduction in case of BL setup makes both CTV2 and CTV1‐2 coverage significantly worse (p=0.0003 and p=0.0044, respectively, McNemar's test), in case of FM setup with the reduced 7 mm margin, the CTV2 coverage is even better compared to BL setup with 10 mm margin (p=0.049, Fisher's exact test). There was no significant difference in CTV1‐2 coverage between BL‐10 mm and FM‐7 mm group (Fig. 6).

Analyzing reasons of CTV2 underdosage in BL‐10 mm group, these can be explained mostly by excessive rectal filling, which can cause prostate movement out of irradiated volume (see Fig. 1(a)). In two cases of underdosed plans, the rectal volume was more than three times larger, in two cases more than two times larger, and in two cases more than 1.5 times larger than volume at initial planning CT. Also the CTV1‐2 underdosage was associated with different rectal filling in planning CT and CBCT, which causes CTV1‐2 movement in anteroposterior direction. In FM‐7 mm group, only one case of CTV2 underdosage can be assigned to excessive rectal volume, which caused rotational movement of CTV2 around apex anteriorly. The remaining five cases of slight CTV2 underdosage can be assigned to intrafractional movement between setup and CBCT acquisition, which was observed via comparison of markers’ position on reference setup images and DRR from CBCT. CTV1‐2 underdosage in FM‐7 mm group was caused by rectal filling in six cases and by intrafractional movement in five cases.

**Table 3:** Number of plans where prostate (CTV2) or seminal vesicles (CTV1‐2) were underdosed. Comparison of bony landmark setup with 10 mm margin (BL‐10 mm), bony landmark setup with 7 mm margin (BL‐7 mm), and fiducial markers’ setup with 7 mm margin (FM‐7 mm).

		*BL‐10 mm*	*BL‐7 mm*	*FM‐7 mm*
CTV2	underdosed	12	27	6
	covered	122	107	171
CTV1‐2	underdosed	3	13	11
	covered	126	116	154

**Figure 7:**
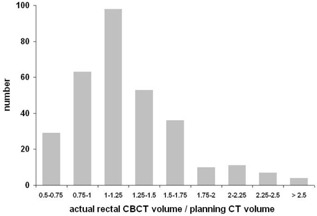
Histogram of actual rectal volumes on CBCTs relative to planning CT volumes.

**Figure 8:**
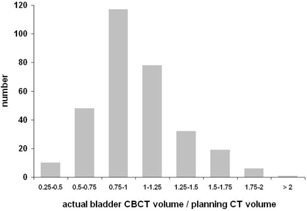
Histogram of actual bladder volumes on CBCTs relative to planning CT volumes.

Rectal volume on CBCT was larger than on planning CT in 70% of all cases. In 7% of all cases, the actual rectal volume was more than two times larger than during initial planning CT (Fig. 7). The systematic underestimation of rectal volume on planning CT could be caused by repeating the planning CT scan in cases of rectal volume exceeding 120 cm^3^ (Fig. 7). Bladder volume on CBCT was smaller than on planning CT in 56% of all cases (Fig. 8).

**Figure 9:**
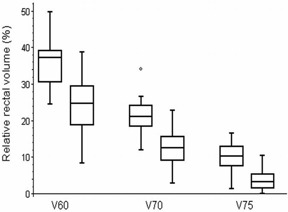
Rectal volumes irradiated with doses higher than 60 Gy, 70 Gy, and 75 Gy derived from treatment plans using 10 mm margin (left) compared to 7 mm margin (right). The box represents the 25th and 75th percentiles, with the central line showing the median value. Two lines extending from the central box of maximal length 3/2 the interquartile range, but not extending past the range of the data. Interquartile range is used to find outliers in data. Outliers are points that are farther than 3/2 times the interquartile range away from the upper and lower quartiles.

**Figure 10:**
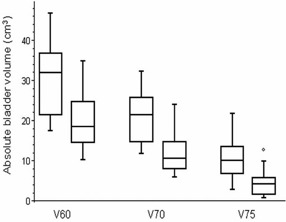
Bladder volumes irradiated with doses higher than 60 Gy, 70 Gy, and 75 Gy derived from treatment plans using 10 mm margin (left) compared to 7 mm margin (right). The box interpretation is the same as in Fig. 9.

Using smaller 7 mm margin, the relative rectal volumes irradiated with a specific doses can be significantly reduced compared to 10 mm margin (Fig. 9): V60 from 36% to 25%, V70 from 22% to 13%, and V75 from 10% to 4%. Absolute bladder volumes irradiated with a specific dose can be significantly reduced, as well (Fig. 10): V60 from 31 cm^3^ to 20 cm^3^, V70 from 21 cm^3^ to 12 cm^3^, V75 from 10 cm^3^ to 4 cm^3^. All the results were statistically significant (p<0.0001, unpaired t‐test).

## IV. DISCUSSION

Feasibility of CBCT‐based dose calculation was evaluated previously by Yoo and Yin.^(10)^ CBCT‐based treatment plans were dosimetrically comparable to CT‐based treatment plans: up to 3% dosimetric error was observed in the plans for the inhomogeneous phantom. Usability of CBCT for dose reconstruction was also investigated by Yang et al.^(11)^ For the static phantom, the doses agreed to within 1%. Based on prostate patient studies, Yang and colleagues concluded that the CBCT can be employed directly for dose calculation for a disease site such as the prostate, where there is little motion artifact. Our comparison of dose distributions computed on inhomogenous anthropomorphic phantom revealed that CBCT‐based plans were comparable to CT‐based plans. Thus, the CBCT can be used to verify treatment delivery retrospectively.^(15)^

A daily cone‐beam CT study of the effect of rectal motion on CTV coverage during prostate radiotherapy was performed by Sripadam et al.^(8)^ CBCT scans were acquired from 15 patients immediately after daily treatment. Daily off‐line electronic portal imaging verification of bony anatomy positioning was carried out, with an intervention level of 5 mm. To obtain the PTV, a 10 mm margin was applied in all directions, except posteriorly where a 7 mm margin was added. A four‐field conformal technique was used with the PTV covered by the 95% isodose. Fields were shaped with multileaf collimators with a penumbra margin of 7 mm. The Sripadam study revealed instances of insufficient CTV coverage occurred in 38% of the fractions delivered to six patients. These only occurred in the upper regions corresponding to the prostate base and seminal vesicles.

Also our results indicate that bony landmark setup is not an optimal choice for prostate radiotherapy. Using IMRT technique with 10 mm CTV‐to‐PTV margin, we observed insufficient prostate coverage in 12 cases distributed over five patients. Most of these cases were caused by excessive rectal volume. Converting into proportion of delivered fractions for purpose of comparison with the Sripadam study, 43% of the fractions delivered to five patients were underdosed. However, the comparison can be affected by lower number of CBCT scans (range four to eight scans) than in the Sripadam study (range 10–16 scans). We observed similar behavior of prostate displacement in cases of prostate insufficient coverage caused by excessive rectal volume — prostate apex seems to be relatively fixed, while the prostate base undergoes rotational movement around apex anteriorly.^7^, ^8^

Hatton et al.^(14)^ assessed the accuracy of the initial CT plan dose‐volume histograms for prostate, rectum, and bladder by comparison to delivered doses determined from CBCT scans acquired immediately following conformal treatment delivery. For the group of 12 prostate patients, daily online implanted fiducial guidance was carried out, with a uniform margin of 7 mm around CTV to determine the PTV. Prostate dose coverage was assessed by the proportion of the CTV fully encompassed by the 95% and 98% isodose lines. Four patients showed marginally compromised CTV coverage by the 95% isodose at all CBCT plans. Hatton and colleagues report 88% of all plans where more than 95% of the prostate volume is covered by the 98% isodose. The Hatton study suggests that margin size of 7 mm is not enough to ensure sufficient prostate coverage at all treatment fractions. This is in agreement with the study by Kasaova et al.,^(16)^ where the intrafraction movement was found to be the reason why it is best not to reduce the margin below 7 mm.

Pawlowski et al.^(13)^ assessed 56 CBCTs for eight prostate cancer patients treated with IMRT. An 8 mm PTV margin everywhere except for 6 mm posteriorly was found adequate with conventional patient positioning using skin tattoos or bony anatomy. Pawlowski and colleagues concluded that the use of intraprostatic fiducials may facilitate significant reduction of planning margins to 4 mm everywhere, except for 3 mm posteriorly.

We considered plans with less than 95% of target volume covered by 95% isodose to be underdosed. The use of bony landmark setup with 10 mm CTV‐to‐PTV margin was a standard practice at our institution. However, this is not enough to ensure sufficient prostate coverage at all treatment fractions. While the margin reduction in the case of BL setup makes the prostate coverage significantly worse, in the case of FM setup with the reduced 7 mm margin, the prostate coverage is even better compared to BL setup with 10 mm margin. There was no significant difference in seminal vesicles coverage between the BL‐10 mm and FM‐7 mm groups.

Recent studies showed that deformation of seminal vesicles relative to intraprostatic markers should be considered.^(17)^ Liang et al.^(18)^ mentioned that the seminal vesicles move independently of the prostate gland and can move significantly more. They assessed the motion of the prostate and seminal vesicles based on multiple daily helical CT during the treatment course, and recommended 3 mm margin for prostate and 4.5 mm margin for seminal vesicles as the minimum values. More recent work by Mutanga et al.^(19)^ found a 5 mm margin for the prostate to be sufficient with daily marker‐based setup corrections. In contrast, an 8 mm margin for seminal vesicles was still insufficient owing to deformations.

In our study, the uncertainty associated with CBCT soft‐tissue definition should be taken into account. In comparison with previously mentioned studies based on helical CT scans,^18^, ^19^ the lower CBCT image quality can be seen in Fig. 1(b). Comparing 2, 3, it can be seen that reproducibility in seminal vesicles delineation is much worse than reproducibility in case of prostate. Therein, the 10 mm margin seems to be suitable for seminal vesicles.

It is still a challenging task to accurately delineate target volumes using CBCT images due to the image quality. The significant differences greater than 3 mm between using fiducial markers with two‐dimensional imaging and using soft tissues with three‐dimensional CBCT imaging for prostate alignment were reported in Barney et al.^(5)^ The difference might be explained by soft‐tissue changes, but the most likely factor resulting in shift differences was that CBCT images of the prostate region were not of a sufficient quality.

Slight patient motion between kV imaging and CBCT acquisition could have occurred, but the Barney study was not designed to resolve this issue. However, a few instances of significant intrafraction motion have been caught in retrospective analysis of fiducials’ position on CBCT. This was not possible without fiducials, because the prostate alone is not visible on planar kV setup images.

Using smaller margin, doses to organs at risk can be significantly lowered. As the volume of rectum receiving ≥60 Gy is consistently associated with the risk of Grade ≥2 rectal toxicity or rectal bleeding,^(20)^ lower complications can be expected. Our results illustrate that rectal V75 can be reduced from 10% to 4%. As mentioned in Mutanga et al.,^(19)^ reducing the V75 by just 5% has a significant impact in the predicted complication probability.

## V. CONCLUSIONS

Reducing of safety margin is not acceptable in case of bony landmark setup, because of the interfractional motion between the prostate and the pelvic bony anatomy. Actual rectal filling can push the prostate out of the irradiated volume. The margin can be lowered from 10 mm to 7 mm in case of fiducial markers setup, which should enable better rectum and bladder sparing. However, deformation of seminal vesicles relative to intraprostatic markers should be considered. Combination of 7 mm margin for prostate and 10 mm margin for seminal vesicles could be a pragmatic conservative approach. Another promising way is the use of CBCT for daily setup, but fiducial imaging requires less daily physician input, is less time‐consuming, and patient exposure to additional ionizing radiation is much lower.

## ACKNOWLEDGMENTS

This work was supported by the Grant Agency of Charles University, Grant GAUK 144210 and by the program PRVOUK P37/09 of Faculty of Medicine in Hradec Králové, Charles University in Prague, Czech Republic.
